# *Lactobacillus reuteri* DSM 17938 and Agave Inulin in Children with Cerebral Palsy and Chronic Constipation: A Double-Blind Randomized Placebo Controlled Clinical Trial

**DOI:** 10.3390/nu12102971

**Published:** 2020-09-28

**Authors:** Andrea A. García Contreras, Edgar M. Vásquez Garibay, Carmen A. Sánchez Ramírez, Mary Fafutis Morris, Vidal Delgado Rizo

**Affiliations:** 1 Departamento de Nutrición y Bienestar Integral, Escuela de Medicina, Instituto Tecnológico y de Estudios Superiores Monterrey Campus Guadalajara, C.P. 4520 Zapopan, Jalisco, Mexico; andrea.garcia@tec.mx; 2 Departamento de Fisiología, Centro Universitario de Ciencias de la Salud, Universidad de Guadalajara, C.P. 44340 Guadalajara, Jalisco, Mexico; mfafutis@gmail.com (M.F.M.); vidalrizo@gmail.com (V.D.R.); 3 Departamento de la Facultad de Medicina, Facultad de Medicina, Universidad de Colima, C.P. 28040 Colima, Colima, Mexico; carmen_sanchez@ucol.mx

**Keywords:** cerebral palsy, *Lactobacillus reuteri* DSM 17938, agave inulin, chronic constipation, children

## Abstract

The main objective was to assess the efficacy of a probiotic (*Lactobacillus reuteri* DSM 17938), a prebiotic (agave inulin), and a synbiotic on the stool characteristics in children with cerebral palsy and chronic constipation. Thirty-seven children with cerebral palsy and chronic constipation were included. The probiotic group received 1 × 10^8^ colony forming unit (cfu) of *L. reuteri* DSM 17938 plus placebo, the prebiotic group received 4 g of agave inulin plus placebo, the synbiotic group received *L. reuteri* DSM 17938 plus agave inulin, and the placebo group received two placebos for 28 days. The probiotic group showed a significant decrease in stool pH (*p* = 0.014). Stool consistency improved in the prebiotic group (*p* = 0.008). The probiotic, prebiotic, and synbiotic groups showed a significant improvement in the history of excessive stool retention, the presence of fecal mass in the rectum, and the history of painful defecation. *L. reuteri* concentration in feces was higher in the probiotic group than in the placebo group (*p* = 0.001) and showed an inverse correlation with stool pH in the probiotic group (*r* = −0.762, *p* = 0.028). This study showed that the use of *L. reuteri* DSM 17938 and/or agave inulin improved the stool characteristics such as the history of painful defecation and the presence of fecal mass in the rectum against placebo in children with cerebral palsy and chronic constipation.

## 1. Introduction

Cerebral palsy (CP) is defined as a group of permanent disorders affecting the development of movement and posture, causing activity limitation, which are attributed to non-progressive disturbances that occurred in the developing fetal or infant brain [1]. The most common gastrointestinal alteration in children with cerebral palsy is constipation [2] at a prevalence of 74% [3]. The normal frequency of defecation in healthy children is from three times a day to once every two days [4], whereas in children with CP, this frequency is reduced from once a week to once every 10 days [5,6,7]. Evidence indicates that dysbiosis of the gut microbiota may also contribute to functional constipation [8]. At present, treatment options for constipation are mainly traditional methods such as purgative therapy and surgery [9]; however, there has been a growing interest in the use of synbiotics, prebiotics, and probiotics. Probiotics are live microorganisms that, when administered in adequate amounts, confer a health benefit to the host [10]. Prebiotics are non-digestible food ingredients that selectively stimulate the growth and/or activity of one or a limited number of bacteria in the colon, thereby benefiting the well-being and health of the host [11]. A synbiotic is a mixture of probiotics and prebiotics that beneficially affects the host [12]. Targeting treatments for dysbiosis with probiotics, prebiotics, and synbiotics improve clinical symptoms, promote the recovery of intestinal flora, and may be a new option that is significantly useful for the treatment of chronic constipation [13,14]. It has been reported in children and adults with functional constipation, treatment with *L. reuteri* DSM 17938 improves defecating frequency [8,15,16]. Treatment with inulin as a prebiotic has shown improved stool consistency [17,18]. Many researchers have suggested the beneficial effect of probiotics in the management of constipation, especially *Lactobacillus* and *Bifidobacterium* [19,20,21]. Therefore, the objective of this study was to assess the effect of a probiotic, a prebiotic, and a synbiotic on the characteristics of stool in children with CP and chronic constipation.

## 2. Materials and Methods

### 2.1. Subjects and Study Design

In a double-blind, randomized controlled clinical trial, children aged 14 to 60 months with CP at levels IV and V of gross motor function as evaluated and classified by a pediatric neurologist were included. All participants presented with chronic constipation according to the Rome IV criteria and were recruited either in the pediatric nutrition and/or neurology outpatient clinic of the Nuevo Hospital Civil de Guadalajara or in the Centro de Rehabilitación Infantil Teletón de Occidente (CRIT) during the period from March 2017 to May 2018. Children were not included when (I) they presented CP of postnatal origin or comorbidities not associated with CP, (II) they were receiving antibiotics, prebiotics, and/or probiotics during the month before the study, and (III) the parents or legal guardians did not agree to participate in the study. Participants were further eliminated when (IV) the child’s parent or legal guardians refused to continue in the study, (V) antibiotics, probiotics, and/or prebiotics (not indicated) were used during the study, (VI) treatment administration had less than 95% compliance, and (VII) hospitalization was required due to external reasons for treatment. The sample size was calculated based on the frequency of defecations using the data published by Indrio et al. [16].

### 2.2. Intervention and Monitoring

The primary endpoints of this trial were the pH, consistency and frequency of the stools. In the study, participants were enrolled by the principal researcher and randomly assigned to a group of intervention by an external person through sealed and opaque envelopes containing a figure representing the group to which the subject was assigned to. During the study period, parents of the children, the researcher who evaluated the follow-up, and the researchers who performed the analysis of the data were blinded (did not know treatment information). The intervention period lasted 28 days. Each participant received the treatment dose in the same form (drops and powder). The probiotic group received 1 × 10^8^ cfu of *L. reuteri* DSM 17938 (Biogaia AB, Stockholm, Sweden) and 4 g of maltodextrin (Ingredion, Guadalajara, Mexico). The prebiotic group received 4 g of agave inulin (Ingredion, Guadalajara, México) and 5 drops of an oil mixture containing both medium chain triglycerides (Numalip, Ciudad de México, México), and sunflower oil (the same mixture used for *L. reuteri* DSM 17938). The synbiotic group received the same dose of *L. reuteri* DSM 17938 and 4 g of agave inulin, and the placebo group received 4 g of maltodextrin and 5 drops of the oil mixture. All products were furnished by the main researcher. Maltodextrin has a similar appearance and taste as inulin. Parents were advised to administer the assigned treatment doses along with food and to keep the drops refrigerated. Changes in a child’s diet such as consumption of food containing probiotics and/or prebiotics, and use of laxatives or dietary fibers were not allowed throughout the study. Parents received two logbooks to record the daily administration of the treatment doses and the stool characteristics (frequency and consistency according to the Bristol Stool Scale). Adverse effects, such as flatulence and abdominal distension, were also recorded. Monitoring was performed through three to four phone calls per week.

### 2.3. Dietary Surveys

Two 24-h recall questionnaires (at basal week and at the end of the study) were performed. Energy, macronutrient, fiber, and fluid intake were analyzed using the Nutrimind software (Vitamex de Occidente S.A de C.V, Guadalajara, Mexico).

### 2.4. Anthropometry

Two observers were standardized to obtain the weight and length. Weight was obtained with the child using a clean diaper on a Health o meter scale HO1524KL (USA) with a precision of 20 g. Height (H) was obtained by measuring knee height (KH) using a segmometer (Rosscraft, Surrey, BC, Canada). This was performed with the knee flexed at a 90° angle, forming a straight line to the heel. Measurements were taken from the heel to the anterior surface of the thigh on the femoral condyles on the least affected side and was obtained using the formula H = (2.69 × KH) + 24.2 [22]. Weight for age and body mass indexes were calculated using the Brooks reference [23].

### 2.5. Stool pH

Stool samples were obtained directly from the participants and placed in sterile jars (Deltalab S.L., Barcelona, Spain) which were kept in a cooler containing ice blocks. Samples were immediately transported to the laboratory and frozen at −80 °C. An aliquot of 1 g of stool was taken and diluted 10 times in Milli-Q water (Agualab Científica, Ciudad de México, México) [24]. After dilution, the stool pH was determined using a Milwaukee MI 150 potentiometer (Milwaukee, Szeged, Hungary), which was calibrated using buffer solutions at pH 7 and 4. The electrode was washed between each sample with cleaning drops (cleaning solution M 10016, Milwaukee, WI, USA) and recalibrated every 20 measurements.

### 2.6. Stool Consistency

Stool consistency was measured using the Bristol Stool Scale [25].

### 2.7. DNA Extraction and Quantitative Real-Time PCR

An aliquot of 25 ± 3 mg of stool sample was dissolved with 300 µL of Milli-Q water, and the sample was homogenized using a vortex. A volume of 50 µL of lysozime (10 mg/mL, Sigma-Aldrich, St. Louis, MO, USA) was added and the sample was left to stand for one h in a water bath at 37 °C. The DNA extraction protocol was performed according to the Zymo Research (Irvine, CA, USA) extraction kit protocol. Real-time quantitative PCR analysis was performed to determine *L. reuteri* in stool. The SYBR Green enzyme and primers for *L. reuteri* 5′-CAGACAATCTTTGATTGTTTAG-3′ and 5′-GCTTGTTGGTTTGGGCTCTTC-3′ [26] were used with the following conditions: pre-amplification 95 °C for 7 min, 45 cycles of amplification comprising of 95 °C for 30 s, 60 °C for 1 min, and 72 °C for 40 s. Melting: 95 °C 0:00; 65 °C 1 min; 95 °C 00:/continuous. Cooling: 50 °C for 30 s. An aliquot of *L. reuteri* DSM 17938 was grown in MRS broth overnight at 37 °C, a sample was counted in a Petroff-Hausser Counting Chamber, Hausser Scientific VWR (Horsham, PA, USA) to obtain 1 × 10^8^ bacteria/mL and to do DNA extraction of this sample. DNA Qubit^®^ (London, UK) fluorometric quantitation was done to verify the amount of bacteria through *L. reuteri* genome molecular weight to do serial dilutions. The curve had seven points corresponding to 1 × 10^8^, 1 × 10^7^, 1 × 10^6^, 1 × 10^5^, 1 × 10^4^, 1 × 10^3^, and 1 × 10^2^ of *L. reuteri*. The calibration curve was plotted with the CT values versus log of the bacteria concentration. 

### 2.8. Statistical Analysis

The Shapiro–Wilk test was performed to determine the distribution of data. Descriptive data were reported in frequencies and percentages, medians, and interquartile ranges (25th and 75th percentiles). For intragroup analysis, Wilcoxon, Cochran’s Q test, and McNemar tests were used. For intergroup analysis, Mann–Whitney U test and Friedman tests were used. The Kruskal–Wallis test was used for intergroup comparisons. Spearman’s correlation coefficients were determined. Intention to treat and relative risk were calculated. The data were analyzed using SPSS version 21 (SPSS Inc., Chicago, IL, USA), and statistical significance was considered with a value of *p* ≤ 0.05.

### 2.9. Ethics and Trial Registration

The study protocol did not put any participant at risk by strictly adhering to the Declaration of Helsinki according to its last correction made during the 64th Annual Assembly organized by the World Medical Association (2013). Informed consent was obtained from each child’s parents or guardian prior to the enrollment. The Bioethics Committee of the Nuevo Hospital Civil de Guadalajara (number 0167/17) also approved the protocol. International registration was obtained at Clinicaltrials.gov with the identifier NCT03117322.

## 3. Results

### 3.1. Subject Characteristics 

**Figure 1:**
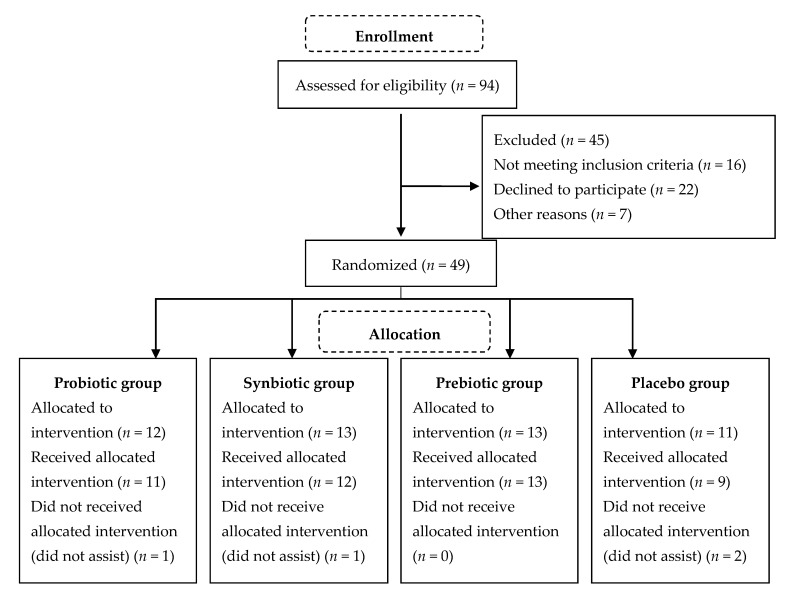

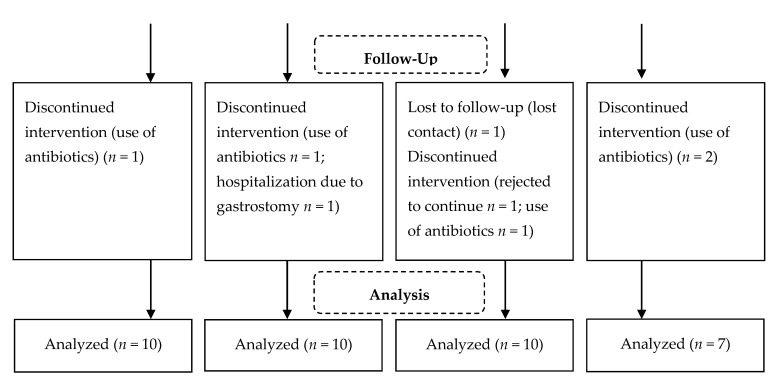
CONSORT flowchart of participant recruitment and study flow.

Forty-nine patients were included in the study, of which 37 completed the treatment (Figure 1). Average age was 37 ± 13 months, and 62% male and 38% female. According to gross motor function, 73% belonged to level V and 27% level IV. Spastic-type CP was the most common type (49%). There were no significant differences in age, weight, height, body mass index (BMI), and weight/age index at the beginning of the study among the four groups (Table 1). 

### 3.2. Rome IV Criteria

According to Rome IV criteria parameters, the probiotic, synbiotic, and prebiotic groups presented significant differences in the history of excessive stool retention, the presence of fecal mass in the rectum and the history of painful defecation at the basal and final weeks (*p* < 0.05). According to the percentage of improvement between the basal vs. the final week of the treatment, the group with the most significant change was the prebiotic with 100% of improvement in history of excessive stool retention, history of painful defecation and 70% in large stool diameter and presence of a large fecal mass in the rectum (Table 2). Relative risk was calculated for each parameter, all of them were significantly lower compared to the placebo group and these results are presented in a supplemental table. The intention-to-treat ratio was 3.

### 3.3. Stool Characteristics

**Figure 2:**
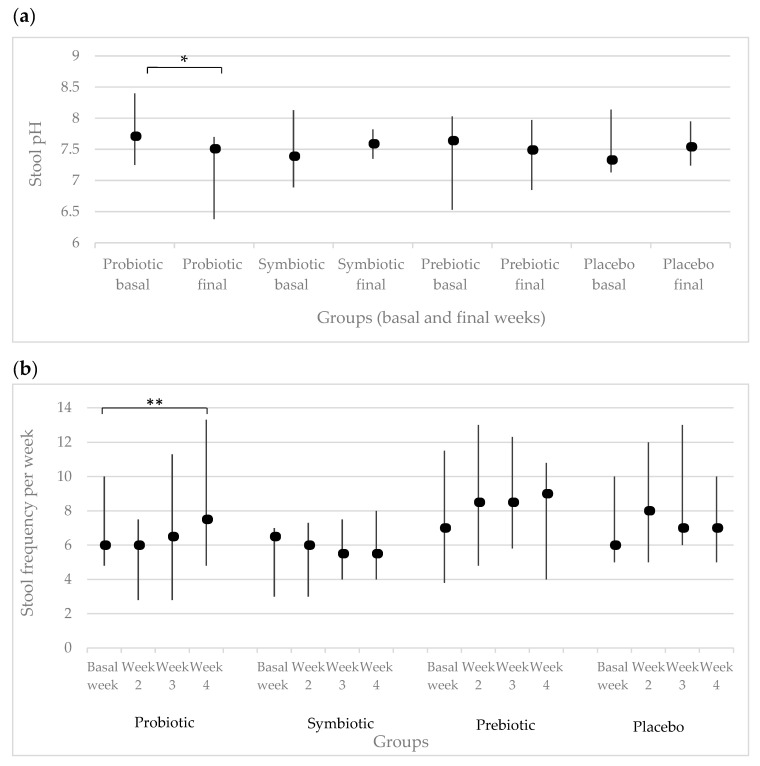
Stool characteristics (median and interquartile ranges). (**a**) Stool pH by group (basal vs final weeks), * *p* = 0.014; (**b**) Stool frequency per week by group, ** *p* = 0.034.

When comparing the stool pH at basal vs. final weeks by group, a significant decrease was observed in the probiotic group (*p* = 0.014) (Figure 2a). Stool frequency per week was analyzed in each group; only the probiotic group showed a significant difference with increased defecation from 6 to 7.5 (*p* = 0.034) (Figure 2b).

Stool consistency was recorded as hard and normal stool. There was a tendency of decreased frequency of hard stool in all groups during the four weeks, except in the placebo group (*p* > 0.05); however, only the prebiotic group showed significant improvement (*p* = 0.008). The frequency of normal stool tended to increase except in the placebo group; only the prebiotic group showed significant improvement (*p* = 0.003). The frequency of hard stool at the basal vs. final weeks of each group was compared to the placebo group. This difference was only significant when comparing the prebiotic to the placebo group (*p* = 0.031) (Table 3).

### 3.4. Dietary Aspects

The average energy intake was 1010 kcal/day, carbohydrates at 136 g/d, fat at 34.5 g/d, protein at 39 g/d, fiber at 8.5 g/d, and fluids at 705 mL/d. There were no significant differences in the energy, macronutrients, fiber, and fluid intake between the basal and final weeks in each group or intergroup (*p* > 0.05).

### 3.5. Gut Microbiota

The presence of *L. reuteri* in stool samples was determined in the probiotic and placebo groups at basal and final weeks. At the end of the study, the concentration of *L. reuteri* was 60 times higher in the probiotic group (5.97 ± 1.7 log_10_ cells/g) than in the placebo group (4.2 ± 1.9 log_10_ cells/g) (*p* = 0.001). Stool pH and concentration of *L. reuteri* in the final week of the probiotic group were significantly inversely correlated (*r* = −0.762, *r*^2^= 0.580, *p* = 0.028).

### 3.6. Adverse Effects

Flatulence was found in 20% of the synbiotic, 40% in the prebiotic, 20% in the probiotic, and 14% in the placebo groups. Abdominal distension was found in 30% of the probiotic and 14% in the placebo groups. There were no significant differences between each group vs. placebo group for each condition. 

## 4. Discussion

This is the first double-blind randomized controlled clinical trial to analyze the efficacy of a probiotic and/or a prebiotic in the treatment of constipation in children with CP. In this study, we have shown that intervention with *L. reuteri* DSM 17938 and/or agave inulin significantly improved stool characteristics in children with CP and chronic constipation. Although some drugs have been proposed for the treatment of constipation in children with CP, such as the study by Imanieh et al. [27] using polyethylene glycol (PEG) and/or motilium, there have been no studies examining the effect of probiotics, prebiotics, and synbiotics in this population.

According to the Rome IV criteria, there was no difference in the bowel movements per week in the study population, this may be because most of the participants (73%) had more than two bowel movements per week at basal week. An interesting finding was that in certain parameters, the synbiotics had less effect than the probiotics and the prebiotics. This may be due to the combination of probiotic and prebiotic was not adequate to show any marked effects and inulin may not be the best substrate for *L. reuteri* DSM 17938. It was reported that the effect of the treatment can vary according to the combination of the synbiotics used [28]. The combination of *L. reuteri* DSM 17938 plus prebiotics in the treatment of constipation has not been investigated; only the combination of probiotics with drugs such as polyethylene glycol and lactulose has been studied without evidence of favorable effects [29,30].

When comparing the intragroup stool frequency among the four weeks, the probiotic group showed a significant difference with an increase of 1.5 bowel movements; however, a difference of two defecations was observed in the prebiotic group, although there was no significant difference due to the lower interquartile ranges in the final week. Some studies on the use of *L. reuteri* DSM 17938 reported significant differences in the stool frequency [8,16]. Ojetti et al. [31] demonstrated that in patients with constipation, the production of methane gas (CH_4_) by the intestinal microbiota, mainly *Methanobrevibacter smithii,* leads to slow intestinal transit and inhibits intestinal motility. In this study, the administration of *L. reuteri* DSM 17938 over a period of four weeks significantly decreased the production of methane gas and improved intestinal motility. Some studies using inulin have reported differences in stool frequency [32,33]. The study by Yu et al. [28] reported significant differences with the use of fructooligosaccharide (FOS) and a mixture of probiotics. Probably, the combination used in our study was not adequate to show significant changes in stool frequency. However, there was a significant difference in stool consistency in the prebiotic group. The effect of inulin on stool consistency could be due to its mechanism of action by stimulating the production of short chain fatty acids, and some metabolites such as lactate, making stool softer and easier to be expelled [32]. Several studies involving inulin have shown improvement in stool consistency [17,18,32,34]. The study carried out by Closa-Monasterolo et al. [17] with a dosage of 4 g inulin per day for six weeks in children with constipation aged two to five years old, demonstrated a soft stool consistency. A double-blind randomized clinical trial demonstrated that prebiotics could soften stool and increase stool frequency [35]. Studies conducted with *L. reuteri* DSM 17938 did not show changes in stool consistency [8,15].

There was a tendency to acidify the stool pH in the probiotic group; this could be attributed to the reuterin produced by *L. reuteri*, an antimicrobial substance that inhibits the adhesion of pathogens to the intestinal epithelium [36]. In the prebiotic group, the stool pH tended to decrease although it was not significant. One of the inulin’s mechanisms of action is the production of short chain fatty acids (butyric, propionic, and acetic acids) from which 80 to 90% are absorbed in the intestine and the rest are excreted through the stool. These short chain fatty acids act to acidify the luminal pH, decrease the growth of pathogenic bacteria, and favor the growth of beneficial bacteria, producing an improvement in intestinal motility [37].

In a previous study involving children with CP and chronic constipation [38], researchers found a low dietary intake for fiber (10 g) and fluid (945 mL), which were similar to the findings in this study, and both were below the optimal dietary requirements. Furthermore, our results are similar to the findings of Caramico-Favero et al. [39] in children with CP and constipation with a dietary fiber intake of 9.2 ± 4.3 g; however, the fluid intake of 457 ± 283 mL is lower compared to our finding. 

After the administration of *L. reuteri* DSM 17938, we observed a 60-fold increase of the probiotic in the stool of the probiotic group compared to the placebo group, indicating that *Lactobacillus* has the ability to remain alive in the gastrointestinal tract and not be digested by enzymes. The inverse correlation between stool pH and concentration of *L. reuteri* indicates that the higher the concentration of *Lactobacillus*, the lower the pH. With this finding, it can be inferred that *L. reuteri* DSM 17938 has the ability to produce organic acids to decrease the luminal pH, which is a mechanism of action against pathogenic bacteria by producing unfavorable conditions in the microenvironment, preventing the adhesion of pathogens to the intestinal epithelium. Consequently, an improvement in intestinal motility occurs and intestinal transit is accelerated through the decrease in CH_4_ [31]. Since flatulence was observed in all the four groups, we cannot confirm if it was due to only one agent or there are other factors involved. Gastrointestinal symptoms such as excess gas and bloating are physiological and related to the intake of dietary fibers and intestinal fermentation [32]. 

One of the strengths of our study is that the participants were well monitored throughout the study by means of telephone calls; in addition, all the participants submitted their logbooks. One of the limitations of our study is the duration of intervention, which may not be adequate to show significant differences in some parameters. A study involving children with CP requires delicate handling because they tend to have recurrent health complications such as pneumonia and bacterial infections, and it is unethical not to treat them with antibiotics. Another limitation is the small sample size. Our results can only be generalized to children with CP with chronic constipation.

## 5. Conclusions

The use of *L. reuteri* DSM 17938 and agave inulin is an effective alternative to improve stool characteristics and constipation in children with CP. *L. reuteri* DSM 17938 improved intestinal motility and acidified stool pH, while agave inulin improved stool consistency, but when analyzed together, they did not show the same effect. Longitudinal studies are crucial to evaluate the effect of probiotic and prebiotic treatments with a longer intervention as well as using different combinations of prebiotics with *L. reuteri* DSM 17938 to determine the best substrate that favors the growth of lactic acid bacteria.

## Figures and Tables

**Table 1:** Baseline characteristics of the study population.

Variable	Groups				*p* *
	Probiotic (*n* = 10)	Synbiotic (*n* = 10)	Prebiotic (*n* = 10)	Placebo (*n* = 7)	
Age (months)	39 (28–51)	42 (29–49)	33 (22–53)	37 (22–45)	0.842
Weight (kg)	10.9 (10.0–13.4)	9.2 (7.9–12.7)	11.0 (8.9–14.2)	9.9 (8.2–11.4)	0.257
Height (cm)	88.1 (84.7–95.8)	89.2 (81.8–93.4)	89.6 (79.8–93.4)	85.5 (80.7–92.5)	0.844
BMI (kg/m^2^)	13.6 (12.6–14.4)	12.7 (11.7–14.5)	13.9 (12.4–15.6)	13.2 (11.4–13.7)	0.309
BMI (Z)	−1.0 (−1.4–(−0.9))	−1.4 (−1.9–(−0.6))	−0.9 (−1.5–(−0.3))	−1.2 (−1.9–(−0.9))	0.337
Weight/age (Z)	−0.2 (−0.9–(−0.0))	−1.2 (−1.7–(−0.0))	−0.3 (−1.0–(−0.9))	−0.9 (−1.5–(−0.2))	0.411

* Kruskal–Wallis test.

**Table 2:** Roma IV criteria for constipation (basal vs. final weeks by group).

Parameter	Groups															
	Probiotic (*n* = 10)				Synbiotic (*n* = 10)				Prebiotic (*n* = 10)				Placebo (*n* = 7)			
	Basal	Final	*p* *	% **	Basal	Final	*p* *	% **	Basal	Final	*p* *	% **	Basal	Final	*p* *	% **
Bowel movements per week																
≤2 times	1	1	1.000	0	5	–	0.063	100	3	–	0.250	100	1	–	1.000	100
>2 times	9	9			5	10			7	10			6	7		
History of excessive stool retention																
Yes	7	1	0.031	85	8	1	0.016	88	7	–	0.016	100	4	2	0.500	50
No	3	9			2	9			3	10			3	5		
History of painful defecation																
Yes	8	–	0.008	100	9	1	0.008	89	7	–	0.002	100	7	3	0.125	57
No	2	10			1	9			–	10			–	4		
Large stool diameter																
Yes	10	5	0.063	50	10	4	0.031	60	10	3	0.016	70	7	5	0.500	29
No	–	5			–	6			–	7			–	2		
Presence of a large fecal mass in the rectum																
Yes	10	2	0.008	80	10	1	0.004	90	10	3	0.016	70	7	4	0.250	43
No	–	8			–	9			–	7			–	3		

* McNemar test; ** Percentage of improvement (basal vs. final weeks).

**Table 3:** Frequency of hard and normal stool by group.

(**a**) Frequency of hard stool.					
**Group**	**Basal Week** ***n* (%)**	**Week 2** ***n* (%)**	**Week 3** ***n* (%)**	**Week 4** ***n* (%)**	*p* *
Probiotic (*n* = 10)	9 (90)	7 (70)	5 (50)	6 (60)	0.097
Synbiotic (*n* = 10)	7 (70)	6 (60)	6 (60)	4 (40)	0.453
Prebiotic (*n* = 10)	8 (80)	5 (50)	3 (30)	3 (30)	0.008
Placebo (*n* = 7)	3 (43)	5 (71)	3 (43)	3 (42)	0.646
(**b**) Frequency of normal stool.					
**Group**	**Basal Week** ***n* (%)**	**Week 2** ***n* (%)**	**Week 3** ***n* (%)**	**Week 4** ***n* (%)**	*p* *
Probiotic (*n* = 10)	1 (10)	3 (30)	4 (40)	4 (40)	0.292
Synbiotic (*n* = 10)	1 (10)	1 (10)	3 (30)	4 (40)	0.266
Prebiotic (*n* = 10)	1 (10)	3 (30)	5 (50)	7 (70)	0.003
Placebo (*n* = 7)	3 (43)	1 (14)	2 (29)	3 (43)	0.564
(**c**) Frequency of hard stool compared to placebo.					
**Group**	**Basal Week**	**Final Week**	**Basal-Final Difference**	*p* ** (Basal-Final vs. Placebo)	
Probiotic	9 (90)	6 (60)	3	0.121	
Synbiotic	7 (70)	4 (40)	3	0.063	
Prebiotic	8 (80)	3(30)	5	0.031	
Placebo	3 (43)	3 (43)	0	-	

* Cochran’s Q test; ** Mann–Whitney U test.
